# A Glossary for Chemical Approaches towards Unlocking the Trove of Metabolic Treasures in *Actinomycetes*

**DOI:** 10.3390/molecules27010142

**Published:** 2021-12-27

**Authors:** Jianye Zhang, Heba Ali Hassan, Usama Ramadan Abdelmohsen, Eman Maher Zahran

**Affiliations:** 1 Key Laboratory of Molecular Target & Clinical Pharmacology and the State & NMPA Key Laboratory of Respiratory Disease, School of Pharmaceutical Sciences & The Fifth Affiliated Hospital, Guangzhou Medical University, Guangzhou 511436, China; jianyez@163.com; 2 Department of Pharmacognosy, Faculty of Pharmacy, Sohag University, Sohag 82524, Egypt; 3 Department of Pharmacognosy, Faculty of Pharmacy, Minia University, Minia 61519, Egypt; 4 Department of Pharmacognosy, Faculty of Pharmacy, Deraya University, Universities Zone, New Minia City 61111, Egypt

**Keywords:** *Actinomycetes*, antibiotics biosynthesis, cryptic genes, elicitors, biological potential

## Abstract

Actinobacterial natural products showed a critical basis for the discovery of new antibiotics as well as other lead secondary metabolites. Varied environmental and physiological signals touch the antibiotic machinery that faced a serious decline in the last decades. The reason was exposed by genomic sequencing data, which revealed that *Actinomycetes* harbor a large portion of silent biosynthetic gene clusters in their genomes that encrypt for secondary metabolites. These gene clusters are linked with a great reservoir of yet unknown molecules, and arranging them is considered a major challenge for biotechnology approaches. In the present paper, we discuss the recent strategies that have been taken to augment the yield of secondary metabolites via awakening these cryptic genes in *Actinomycetes* with emphasis on chemical signaling molecules used to induce the antibiotics biosynthesis. The rationale, types, applications and mechanisms are discussed in detail, to reveal the productive path for the unearthing of new metabolites, covering the literature until the end of 2020.

## 1. Introduction

The phylum *Actinobacteria* is one of the most diverse phyla of the Gram-positive bacteria, from which *Actinomycetes* are widely distributed in different natural habitats, including soils, lakes and oceans. Different *Actinomycetes* are also found in nature associated to many invertebrates and plants [1,2]. From the “antibiotic golden era”, the period from 1940 to 1960 of antibiotic discovery by the phenotypic screening of soil microorganisms, *Actinomycetes* have been recognized as fruitful sources of diverse secondary metabolites (SMs) [3,4]. A considerable number of bioactive natural products have been detected from microbial origin, in which micro-organisms have assumed a potential role in strengthening the field of natural product discovery in the last decades [5]. Although products from a natural origin have a proven potency for the eradication of infectious diseases [6], antimicrobial resistance has seriously raised and has been recognized as a threat to humankind, with the prediction of ~10 million deaths by 2050, if no action is applied [7]. Even with the average of 1000 new natural products, that have been discovered each year over the last two decades, the frequent rediscovery of already known compounds continues to be a key bottleneck in the field [8]. Additionally, recent advancements in the genome sequencing of *Actinomycetes* have shown “an innovation gap” between biosynthetic potential and isolated bioactive SMs [9,10]. The genomes of these *Actinomycetes* generally harbor many biosynthetic gene clusters (BGC) that encode paths for the creation of SMs, but with a plethora of silent genes of yet unobserved molecules [11]. The genome analysis revealed that these SMs include non-ribosomal peptides and polyketides, which harbor the enzymes non-ribosomal peptide synthetases (NRPSs) and polyketide synthases (PKSs), included in their biosynthesis, respectively [12]. It is reported that the exploration of these silent genes (which encode and produce known BGCs upon their awakening) have the ability to assist in the exploration of the rare molecules that are generally hard to be obtained [13]. Such a scenario has encouraged scientists to discover various techniques and strategies to de-silence these BGCs, trigger their expression and identify new natural products and molecular scaffolds [14]. The techniques involve the “one strain many compounds” approach (“OSMAC”), co-cultivation, ribosome engineering, as well as utilizing molecular, biological and chemical elicitors [15]. The co-cultivation has already been applied for the production of foods, food additives, enzymes and chemicals, but is not widely used for antibiotic production. On the other hand, the manipulation of fermentation conditions, or “one strain many compounds” “OSMAC” [16], represents a potential strategy for activating poorly expressed metabolic pathways, with the disadvantage of producing a low yield [17].

Accordingly, many studies have focused on alternatives that have the ability to produce secondary metabolites with rational amounts suitable for performing further analysis. Sensitization of the microbial cells with external elicitors, is a well-recognized strategy, using small organic molecules in their minimal effective concentrations for inducing silent BGCs in *Actinomycetes* cultures to produce suitable amounts of SMs [18]. Consequently, distinct responses (altered metabolite profile) are created inside the microorganisms [14], expressed in the enhanced production of stress–response associated compounds [19], and trigger the biofilm formation and/or the modified expression of virulence [15]. Such elicitors include antibiotics, histone deacetylase inhibitors, rare earth elements, autoregulators as well as minute amounts of heavy metals [20], all of which will be discussed in details, in the present paper.

**Figure 1:**
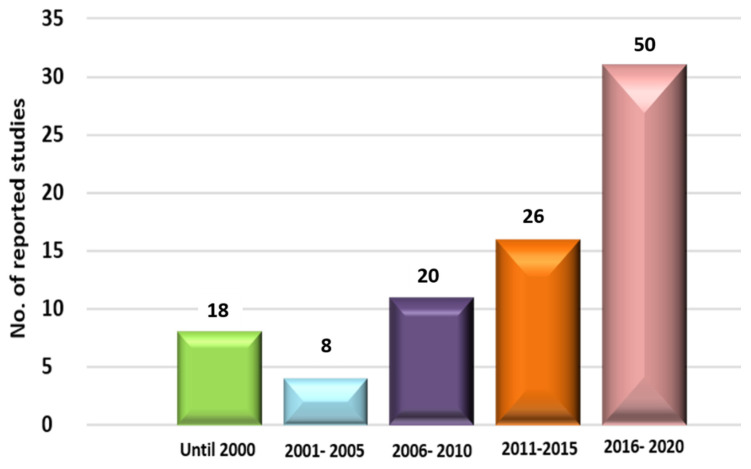
Secondary metabolites elicited from different *Actinomycetes* according to the year of publication.

Although there is a number of reviews covering the elicitation in *Actinomycetes* [18,21], our review is considered as specific for chemical approaches and also complementary to the first review published [15] that covered the period until the end of 2014. The present review gives a special concern to the elicited SMs, which exhibited strong biological activities, while the data covering all the elicited ones, even those that have not yet been tested, are enumerated in the Supplementary Materials. Our review signifies the new approaches that are currently being developed, to propose novel antimicrobials, with a distinct focus on chemical elicitors and how their regulatory mechanisms have evolved in the context of ecology and genomic perspectives. The review covers the published data until the end of 2020, with the data being analyzed and grouped into year intervals according to the collected studies (Figure 1). The data have been evaluated through several search engines, such as Pubmed^®^, Science direct^®^, SciFinder^®^, ISI^®^, Scopus^®^ and Google Scholar^®^, using “chemical elicitor” as the search key word. All detected secondary metabolites from different classes of elicitors are listed in the Supplementary Materials (Table S1).

## 2. Specific Chemical Elicitors

### 2.1. Antibiotics and Their Biosynthetic Intermediates

#### 2.1.1. Antibiotics at Sub-Inhibitory Concentrations

**Figure 2:**
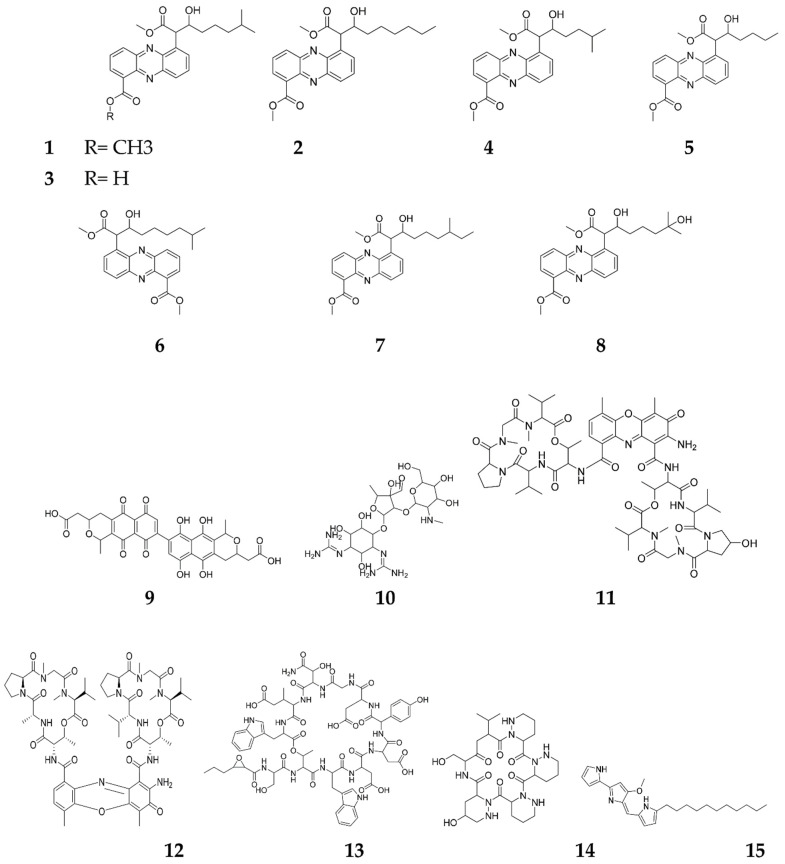
Secondary metabolites elicited by sub-inhibitory concentrations of antibiotics.

Transcriptomic studies have revealed that sub-inhibitory concentrations (SICs) of antibiotics can stimulate many regulators, especially those belonging to the PAS-LuxR family [22], which are classified under the quorum sensing regulators [23]. These PAS-LuxR family members comprise of PimM, NysRIV and PteF regulators of pimaricin, nystatin and filipin biosynthesis, respectively [24]. As reported, SICs as low as 1% of minimum inhibitory concentration (MIC) values, can trigger many phenotypic changes, such as the induction of biofilm formation, creating a virulence factor, increasing bacterial motility or activating the cryptic natural products biosynthesis [11]. In a marine *Streptomyces* spp., streptophenazine A (**1**) has been elicited with SICs of either bacitracin or tetracycline, to attain elevated titers by 2.2-fold at 0.1 μg/mL concentration [11]. Tetracycline also elicited streptophenazines E–H (**5**–**8**), and enhanced streptophenazines A–D (**1**–**4**), while bacitracin induced streptophenazine H (**8**) with titers elevated between 2.6 and 10.7-fold [25]. The SIC of monensin induced cryptic isonitrile antibiotic SF2768 in the *S. griseorubiginosus* strain 574, while lincomycin (**9**) enhanced streptomycin (**10**) production in *S. griseus*, from 40–100 mg/mL [26] via binding to the ribosomes of Gram-positive bacteria and modifying the translational apparatus [27]. Additionally, lincomycin at one tenth of its MIC, enhanced the expression of the pathway-specific regulatory gene actII-ORF4, belonging to the SARP family, which is the main activator of ACT (**11**) biosynthesis in the enzyme gene cluster [28]. Moreover, chloramphenicol triggered a 1.5- to 6-fold enhancement in the biosynthesis of actinomycin D4 (**12**), calcium dependent antibiotics (CDAs, **13**) and piperidamycin (**14**) in *Streptomyces* sp. [29], all of which exhibited a wide range of antimicrobial activities [30,31]. Interestingly, actinomycin D triggered the extrinsic pathway of apoptosis and acted synergistically with RG7787 to cause prominent tumor regression in mice [32]. It has been clinically tested for Wilms tumor of the kidney, rhabdomyosarcoma and trophoblastic tumors as well as down-regulating the expression of the stem cell transcription factor, Sox-2; a breast cancer fighter [33]. More interestingly, the SIC of actinomycin D specifically stimulated a p53-dependant transcription factor, which enhanced its killing power of p53 human tumor cells [34], and also prevented the Coxsackie B3 virus and HIV-1 [12,13], making actinomycin D one of the most talented candidates for medicinal development purposes [35]. Furthermore, SICs of some ribosome-targeted antibiotics, such as thiosteptone and spectinomycin, can exert a 4-fold growth in undecylprodigiosin (RED, **15**) production in *S. coelicolor* M145 [36], which attains a wide range of antimycotic, antiprotozoal and larvicidal activities (Figure 2) [37].

#### 2.1.2. Antibiotics as Autoregulators of Their Own Biosynthesis

**Figure 3:**
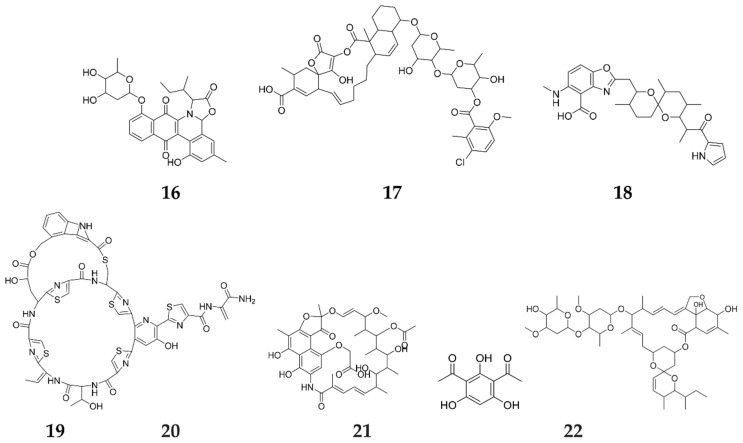
Secondary metabolites elicited by autoregulation from the same regulators.

Recent studies have focused on the role of atypical response regulators (ARRs) that are included in the antibiotic biosynthetic pathways, such as the cluster-situated regulator (CSR) activator JadR1, an OmpR-type ARR of *S. venezuelae*, included in the biosynthesis of jadomycin B (JdB, **16**) [38]. Generally, antibiotics exert their regulatory effects via CSRs that are linked to BGCs or pleiotropic regulators (regulators that control the biosynthesis of multiple SMs) [3]. JdB was found to specifically interact with JadR1 and control its own production in *S. venezuelae* in a highly organized feedback regulatory mechanism [39]. The feedback regulatory mechanism is best expressed by JadR∗, a TetR family regulator that responds to the JdB intermediates [2,3-dehydro-UWM6 (DHU) and dehydrorabelomycin (DHR)] [24]. This indicates that antibiotics and their biosynthetic intermediates have the ability to share a role as ARRs through the modulation of their own biosynthesis. Additionally, ChlF1, which is also a TetR family regulator included in chlorothricin (**17**) biosynthesis in *S. antibioticus*, was found to split from its target promoters in the occurrence of chlorothricin with its biosynthetic intermediates (demethyl salicyloyl chlorothricin and deschloro-chlorothricin), followed by the interaction with this regulator [40]. Moreover, CalR3, which is a TetR family regulator involved in the calcimycin (**18**) biosynthesis of *S. chartreusis* NRRL 3882, responds to calcimycin and its biosynthetic intermediates (dimethyl-salicycloyl chlorothricin and deschloro-chlorothricin) [40]. Furthermore, NosP regulates the production of nosiheptide (NOS) (**19**) in *S. actuosus*, in response to both nosiheptide as well as its biosynthetic intermediates [41]. Nosiheptide exhibited a strong anti-mycobacterial activity against *M. avium* and *M. intracellulare*, with obtained MICs in the range of 0.024 to 1.56 μg/mL [42]. Apart from *Streptomyces*, the Actinomycete *Amycolatopsis mediterranei* plays a role as an extracellular signaling molecule regulating rifamycin B (**20**) export in a feedback mechanism [41], while 2,4-diacetylphloroglucinol (2,4-DAPG, **21**) and its biosynthetic intermediate monoacetylphloroglucinol can regulate the biosynthesis of 2,4-DAPG in *Pseudomonas fluorescens* (Figure 3) [43].

#### 2.1.3. Antibiotics as Autoregulators of Other Biosynthetic Pathways

One of the important examples of this type of autoregulator is JadR2, a common TetR family regulator that modulates JdB biosynthesis in *S. venezuelae* via the direct repression of jadR1, which is sensitive to JdB end products [44]. Interestingly, JdB combines with apramycin, hygromycin B or kanamycin, then modulates the receptor DNA-binding activity of AvaR2, leading to an enhancement of the production of the macrocyclic lactone avermectin (**22**) [3], which attains significant anthelmintic and insecticidal/acaricidal activities (Figure 3) [45].

#### 2.1.4. Antibiotics as Cross-Regulators of Other Antibiotics Biosyntheses

**Figure 4:**
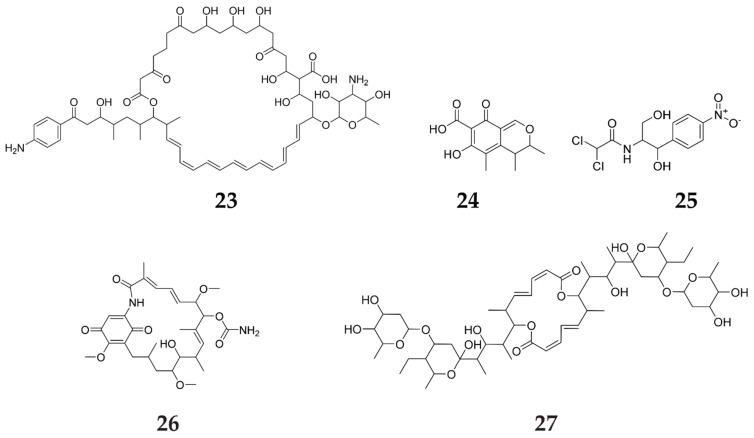
Secondary metabolites elicited by cross-regulation from other regulators.

RedZ, a CSR of the red gene cluster, was found to modulate the production of RED, ACT as well as CDA in *S. coelicolor* [46]. The same idea was discussed in a study that reported that FscRI (the candicidin CSR) was detected to regulate the overall biosynthesis of both candicidin (**23**) and antimycin (**24**), the end products of a different BGC in *S. albidoflavus* S4 [47]. In the same context, JadR1 was observed to control JdB as well as chloramphenicol (**25**) production in *S. venezuelae* [39], while GdmRIII proved to enhance geldanamycin (**26**) and elaiophylin (**27**) in *S. autolyticus* CGMCC0516 [48]. It is worth mentioning that geldanamycin is a unique antibiotic exhibiting virucidal, ischemia protective and anti-neoplastic activities (Figure 4) [49].

### 2.2. Antibiotic Remodeling Compounds (ARCs)

**Figure 5:**
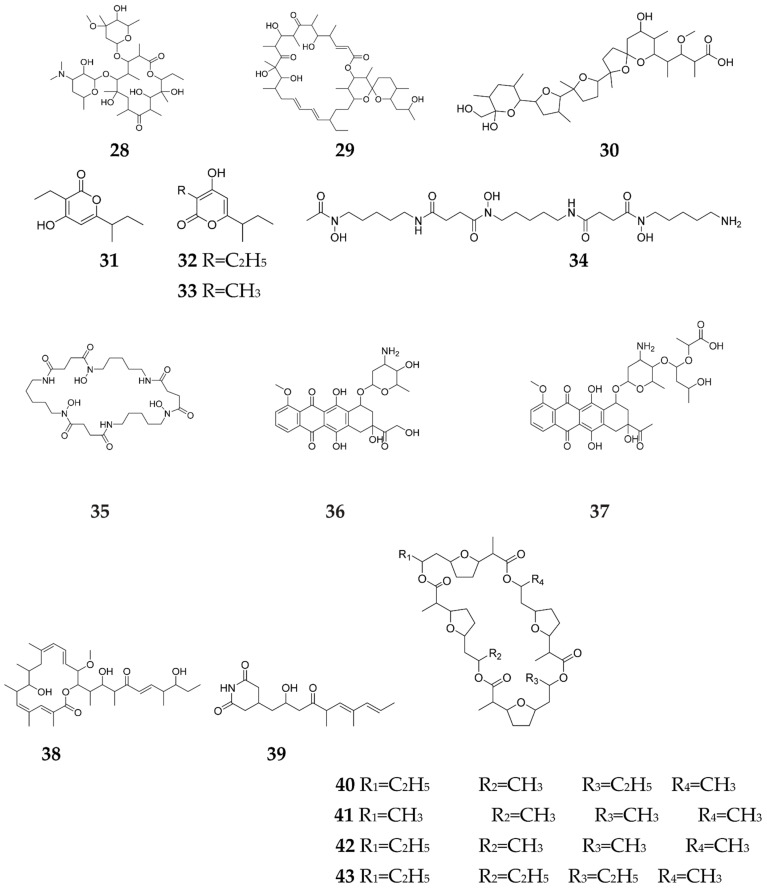
Secondary metabolites elicited by antibiotic remodeling compounds (ARCs).

A family of fully synthetic chemicals with the ability to modify the fatty acid biosynthesis, known as antibiotic remodeling compounds (ARCs), have been recently investigated and observed to change fatty acid pools through countering the key biosynthetic enzymes as FabI [50], and displaying a superior current of acetyl-CoA for the antibiotic biosynthesis [50]. Notably, a large number (>30,000) of ARCs were screened to understand the regulatory network by using chemical perturbation of secondary metabolism [51]. Regarding the ability to remodel ACT production, the above mentioned screening yielded 19 compounds, of which ARC2, ARC3, ARC4 and ARC5 were the most active [52]. ARC2, as the top active, can encourage the buildup of elevated levels of unsaturated fatty acids in *Streptomyces* sp., which enhanced the production of erythromycin (**28**), oligomycin (**29**), monensin B (**30**) and ACT [53], and achieved a three-fold increase in germicidins A-C (**31**–**33**) [29]. Moreover, it enhanced the production of the siderophore, desferrioxamine B (**34**) and E (**35**) in *S. pristinaespiralis*, and the antibiotics doxorubicin (**36**) and baumycin (**37**) in *S. peucetius* [52], all of which attain powerful anticancer properties [52]. Interestingly, ARC2, ARC3, ARC4 and ARC5 are referred to as the “ARC2 series” for antibiotic–remodeling compounds, as they had similar structures and stimulated blue pigmentation during growth on a solid medium. They can reduce yields of the prodiginines and enhance the production of two other *S. coelicolor* secondary metabolites and germicidin ~3-fold; whereas yields of the daptomycin-like calcium-dependent antibiotic (CDA) were reduced ~2-fold [54]. Accordingly, the ARC2 series can therefore pleiotropically remodel secondary metabolism in *S. coelicolor* [54]. On the other hand, ARC6 elevated the levels of ACT in *Streptomyces* via the same mechanism, but with limitation to *S. coelicolor*, which indicates that it acts more as a species–specific synthetic signal [51]. Recently, a chlorinated analog of ARC2, named Cl-ARC, was employed to trigger the expression of CBGs in an unbiased screening regimen [52]. This resulted in the elicitation of three rare and valuable antibiotics: oxohygrolidin (**38**) (antifungal and insecticidal [55]) from *S. ghanaensis* ATCC 14672, 9-methylstreptimidone (**39**) (anti-inflammatory, antiproliferative and antibacterial [56]) from *S. hygroscopicus* ATCC 53653 and dynactin (**40**), as well as the known antibiotics nonactin (**41**), monactin (**42**) and trinactin (**43**), from WAC0256 [57]. All of them, especially dynactin, showed motility impairing potency against *P. viticola, Phytophthora capsici* and *Aphanomyces cochlioides* zoospores, which indicates non-specific activities toward peronosporomyctes (Figure 5) [58].

### 2.3. Histone Deacetylase Inhibitors (HDACIs)

HDACs are members of a large family of epigenetic drugs that affect histone deacetylation, cause apparent changes in the chromatin structure and attain approval for treating serious cases, such as lymphoma and myeloma [47]. They catalyze many other deacylation reactions, such as demalonylation, decrotonylation or desuccinylation, besides possessing many critical enzyme-independent functions [59,60]. They are classified into four classes: class I (HDAC1, 2, 3 and 8); class II [further sub-grouped into class IIa (HDAC4, 5, 7 and 9) as well as class IIb (HDAC6 and 10)]; class III (HDAC11) and class IV (SIRT1-7) [61]. On the other hand, histone deacetylase inhibitors (HDIs) block these HDAC enzyme activities and inhibit their deacetylation reaction. They bind to the zinc ion inside the catalytic sites of the enzymes, leading to the obstruction of the substrate admission to these sites, thus blocking their action. They include hydroxymates, such as suberoylanilide hydroxamic acid (vorinostat), LBH589 (panobinostat), TSA (trichostatin A) and PXD101 (belinostat) [62,63]. Short-chain fatty acids, such as sodium butyrate (SB) and valproic acid (VPA), prohibit class I and IIa HDACs, while benzamides, such as MS275 (entinostat), and depsipeptides, such as FK228 (romidepsin), prohibit some of the class I HDACs [62,63].

Their main mechanism of activating BGCs is directed towards three HDAC-like genes, which transcriptionally control the biosynthetic pathways in several *Streptomyces* strains, resulting in the modification of the nucleoid structure [64] and enhancement of antibiotic production [18]. SB and VPA (25 mM) have the ability to elicit ACT, as well as five other cryptic pathways with deoxysugar synthetase, hopanoids, sesquiterpene cyclase, germicidin and coelibactin in *S. coelicolor* A3 strain M145 [64].

### 2.4. Hormone-like Signaling Molecules (Autoregulators)

Hormone-like signaling molecules are low molecular weight compounds that can enhance antibiotic production, and/or induce many morphological differentiations at nanomolar concentrations [65]. The first and most common identified autoregulator is the A-factor that greatly induces streptomycin production in *S. griseus* [66]. In general, autoregulators enhance SMs production via passive diffusion into the cells, accumulation beyond the critical concentration [15] as well as targeting the regulatory genes for “cluster-situated regulators” (CSRs), which are linked to antibiotic BGCs [67]. By identifying a total of 24 autoregulators from more than 12 *Streptomyces* species, they can be categorized into 5 major categories: γ-butyrolactones (GBLs), aromatic furans (AFs), γ-butenolides, PI factor and *N*-methylphenylalanyl-dehydrobutyrine diketopiperazine (MDD) [21]. 

### 2.5. Gamma-Butyrolactones (GBLs)

**Figure 6:**
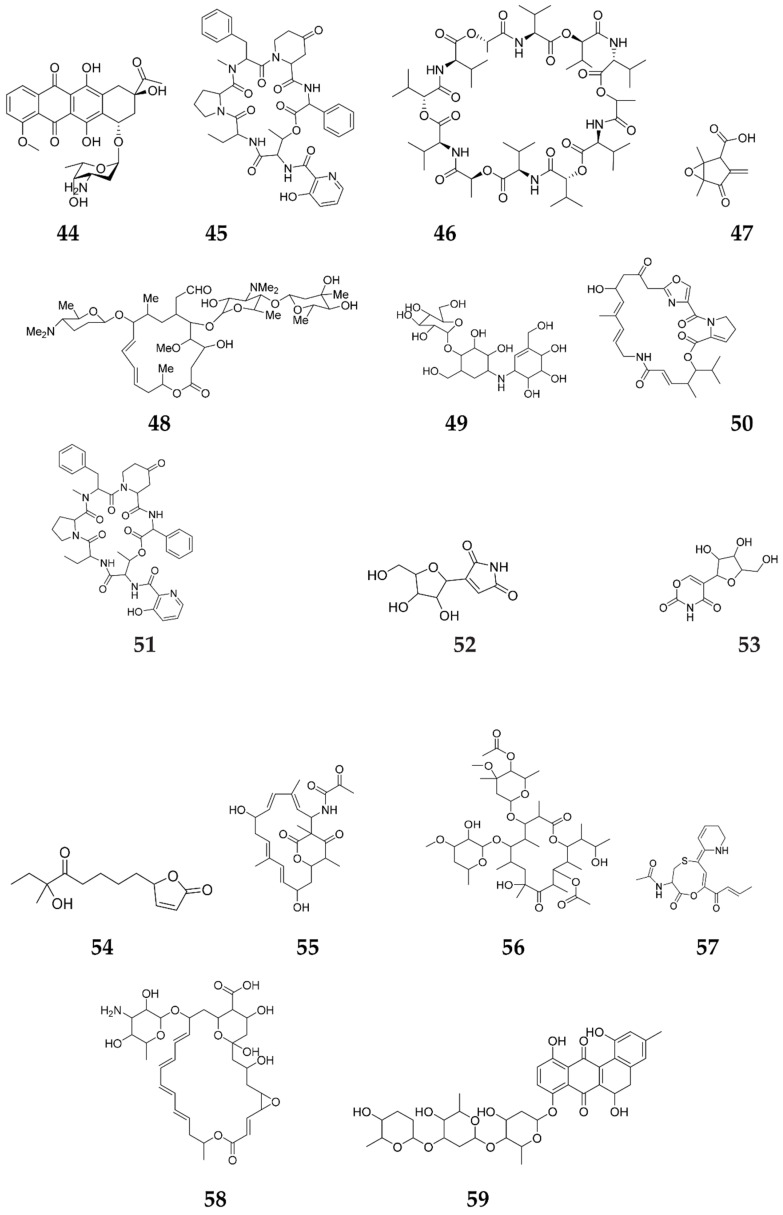
Secondary metabolites elicited by different autoregulators.

Gamma-butyrolactones involve 19 different members, comprising of the A-factor from *S. griseus*, 8 butenolides (SCB1-8) from *S. coelicolor*, 5 virginiae butanolides (VBs A–E) from *S. virginiae*, IM-2 from *S. lavendulae*, methylenomycin factor (MMF) from *S. coelicolor*, factor 1 from *S. viridochromogenes*, as well as 3 Graefe’s factors from *S. bikinensis* and *S. cyaneofuscatus* [3,67]. Formerly, the diastereomeric 4,5-dihydroxydecanoic acid-4-lactones induced anthracycline (**44**) in blocked mutants of *S. griseus* 4 [68]; however, recently, they have been used to induce the biosynthesis of virginiamycin (**45**) in *S. virginiae* [69], auricin (**46**) in *S. aureofaciens* [70] and methylenomycin A (**47**) in *S. coelicolor* A3(2) [71]. Yet, the main obstacles of using exogenous GBs as a convenient strategy to improve antibiotic production in *Streptomyces*, is their nanomolar concentrations, making it difficult to collect enough amounts of antibiotics for their structural elucidation and applications (Figure 6) [72]. The most important GBL, A-factor, has gained much interest with a chief role in regulating streptomycin (**10**) biosynthesis [73], serving as a pleiotropic regulator, while other synthetic A-factor-like regulators induce the biosynthesis of many other antibiotics as virginamycin, valinomycin [18] and methylenomycin (Mm) in different strains of *S. coelicolor* [74]. Recently, the easily accessible A-Factor analog, 1,4-butyrolactone (1,4 B) at 1 mM, has been proven to elicit bitespiramycin (**48**) and validamycin A (**49**) (Figure 6) biological titers in different strains of *S. coelicolor* by 29% and 30%, respectively [75]. This is considered as highly valuable, since naturally produced bitespiramycin attains low titers with limited application and industrialization [75]. The mechanism of the A-factor inducing action was correlated to the accumulation of the two enzymes, methylmalony-CoA and acetyl-CoA, as a direct reason for enhancing bitespiramycin biosynthesis [75]. Concerning virginiae butanolides, five VBs were identified from the culture broth of *S. virginiae* as the key elicitors of virginiamycin [76], with a focus on Virginiae butanolide-C (VB-C), which achieved the maximal production of virginiamycins M (**50**) and S (**51**) in *S. virginiae* (Figure 6) [77]. On the other hand, IM-2 significantly elevated the titers of the nucleoside antibiotics, showdomycin (**52**) and minimycin (**53**), in *S. lavendulae* [78] and enhanced methylenomycin furans biosynthesis from *S. coelicolor* [74].

### 2.6. Aromatic Furans

The first and more common identified aromatic furan was hydroxymethylfuran, which enhanced the methylenomycin genes in *S. coelicolor* for the production of methylenomycin A [74]. The second aromatic furan was 7ae, an enzyme inhibitor of phosphopantetheinyl transferase, which is included in the activation of the acyl carrier protein of fatty acid biosynthesis, resulting in the production of ACT in cultures of *S. coelicolor* [79]. 

### 2.7. γ-Butenolides

The most common butenolides signaling molecules include the avenolide from *S. avermitilis* [80], two butenolides (SRB1 and SRB2) from *S. rochei* [81], four butenolides from *S. albus* J1074 and three butenolides (SAB1-3) from *S. ansochromogenes* [39]. Of special consideration is the avenolide (**54**) that is compulsory for triggering avermectin biosynthesis in *S. avermitilis* [80]. The gene cluster responsible for avermectin biosynthesis harbors three genes (avaR1-3) encoding a GBL receptor homologs, as well as two genes (Aco and cyp17) encoding an acyl-CoA oxidase and a cytochrome P450 hydroxylase, respectively [82]. Moreover, the addition of the GBLs has been used for eliciting the anticancer, lankacidin (**55**) [83], as well as lankamycin (**56**) in *S. rochei* [84]. Using the screening approach, quorum sensing (QS), the first identified butenolide, SCB1, was found to promote ACT production in *S. coelicolor* via binding to its known receptor, ScbR [85]. In addition, SCB1–3 and five other novel GBLs, named SCB4–8, obtained from the genetically engineered strain *S. coelicolor* M1152, were highlighted as valuable elicitors of coelimycin (**57**) (Figure 6) [86].

### 2.8. PI Factor

A good enhancer of pimaricin (**58**) in *S. natalensis*, named PI factor, 2,3-diamino-2,3-bis(hydroxymethyl)-1,4-butanediol, has been detected from *S. natalensis*, and was found to exert its eliciting action at very low concentrations [87]. Pimaricin is a glycosylated polyene, with powerful and promising antiviral, antibacterial and antifungal potencies [88].

### 2.9. N-Methylphenylalanyl-dehydrobutyrine Diketopiperazine (MDD)

The cell-free extracts of a landomycin E-synthesizing strain, *S. globisporus* 1912-2, were shown to harbor a low molecular weight *A*-factor analog, which re-established the landomycin E (**59**) and streptomycin biosynthesis and sporulation of the defective mutants in *S. globisporus* 1912-B2 and *S. griseus* 1439, respectively [89].

### 2.10. Metabolic Signals: GlcNAc and Siderophores

Metabolic signals are prevalent minute molecules controlling antibiotic biosynthesis and morphological differentiation in *Streptomyces* sp., when directly applied to cultures, and are considered part of primary metabolism [90]. The most common one is *N*-acetylglucosamine, that is released from the cell wall during autolysis by the action of chitinases, and is considered as a major food source for *Streptomycetes* Spp. Some other signaling molecules that are still poorly understood are strain-specific peptides (such as the small peptide goadsporin) as well as the protein factor C [67], in addition to glutamate, which is favored over GlcNAc by *S. coelicolor* [91]. It has been reported that metabolic signals act via a signaling cascade, from an extracellular signaling nutrient towards the enhancement of antibiotic production [21]. A related study investigated the effect of GlcNAc on the metabolic profiles of nine sponge-derived *Actinomycetes*, from which only *Micromonospora* sp. RV43, *Rhodococcus* sp. RV157 and *Actinokineospora* sp. EG49 revealed changes in their metabolic profiles [92]. The elicitor induced the bioformation of 3-formylindole (**60**) and guaymasol (**61**) in *Micromonospora* sp. RV43, bacillibactin (**62**) and surfactin lipopeptide 17 (**63**) in *Rhodococcus* sp. RV157 as well as the poorly expressed new metabolites, Actinosporins E-H (**64**–**67**) in *Actinokineospora* sp. EG49 [93]. These results highpoint the use of NMR fingerprinting to perceive fluctuations in metabolic profiles, following the addition of the pleiotropic regulator, GlcNAc, which suppresses metabolites, induces new metabolites and also increases the production of minor compounds [92]. Recently, the HR–ESI–MS technique was applied for dereplication studies, in which G-2N (**68**) and saptomycin F (**69**) were dereplicated, while the antitrypanosomal new compounds fridamycin H (**70**) fridamycin I (**71**) and Actinosporin C, D, and G (**72**–**75**) were isolated from the solid culture of the sponge-associated Actinomycete, *Actinokineospora spheciospongiae* sp. nov. [72]. Moreover, five kigamicin derivatives (**76**–**80**) were also elicited from the fermentation extract of *Amycolatopsis alba* DSM44262Δ*abm9*, after the introduction of 25 mM GlcNAc [94]. From them, Kigamicin D was highlighted as a powerful antitumor that inhibited the growth of various mouse tumor cell lines at IC_50_ of about 1 µg/mL [95]. 

**Figure 7:**
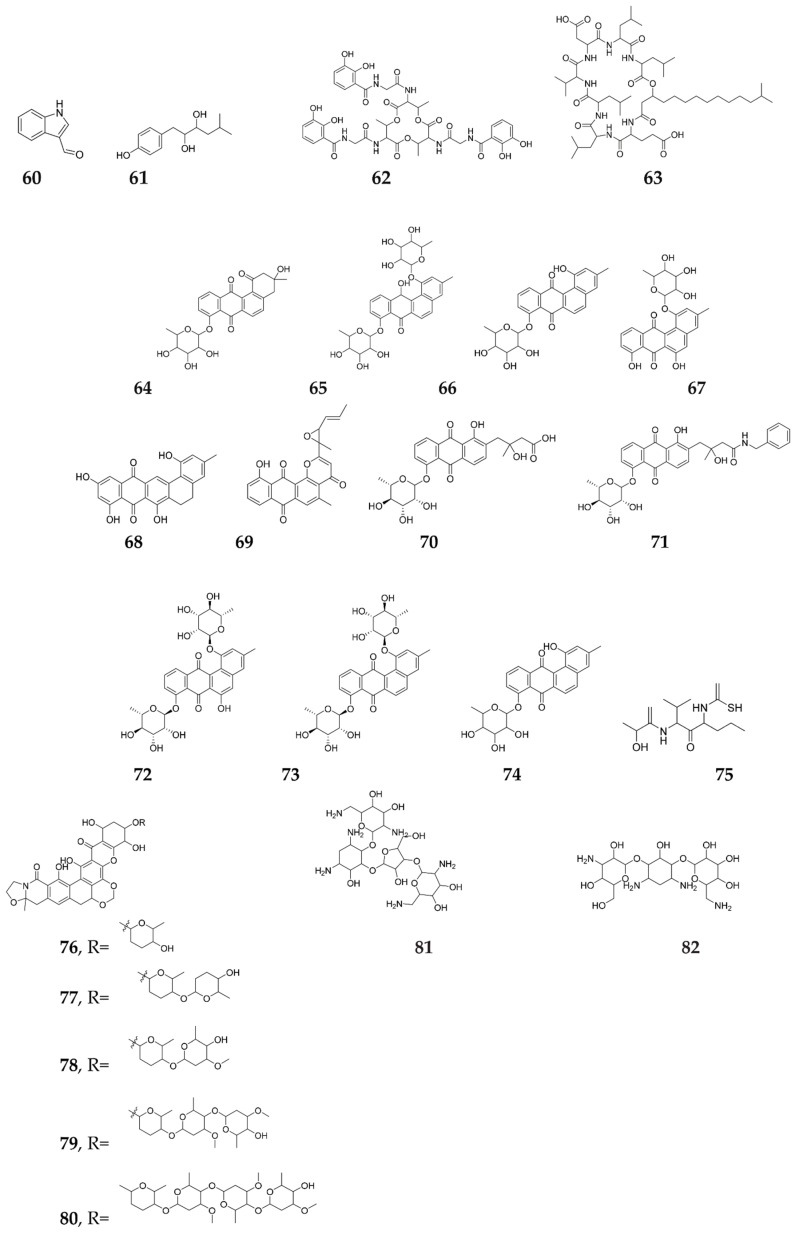
Secondary metabolites elicited by metabolic signals (GlcNAc and siderophores).

Interestingly, desferrioxamine is a fungal growth factor and one of the most common siderophores among terrestrial *Actinomycetes*, and the original supplier of the amount of iron available to the receiving strain included in the binary interaction [96], which indorses developmental features, such as sporulation and SMs production. Desferrioxamine E stimulated the growth, cell differentiation and enhancement of streptomycin, neomycin (**81**) and kanamycin (**82**) production (Figure 7) in *S. tanashiensis* [96]. Interestingly, the *S. coelicolor* mutant defective in desferrioxamine biosynthesis (KY1), showed impaired growth and development on Bennett’s/glucose solid medium, an impairment that is probably due to an iron deficiency and confirmed the previous findings [97]. The KY1 suggested that the bacteria have other ferric uptake system(s), combined with speculating that another peptidic siderophore, that are termed coelichelin and existed in *S. coelicolor* A3(2) [98]. Both *S. coelicolor* and *S. tanashiensis* were found to grow and develop well on Bennett’s medium supplied with maltose, which further suggested that the siderophore production in *Streptomyces* strains is under complex regulation that links to carbohydrates in addition to ferric limitation [96]. 

### 2.11. Rare Earth Elements (REEs)

**Figure 8:**
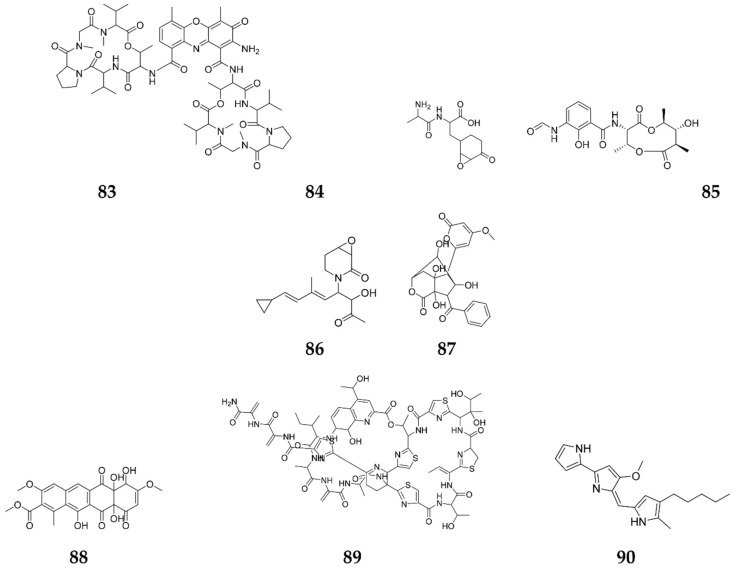
Secondary metabolites elicited by rare earth elements (REEs), heavy metals and organic solvents.

One of the most interesting eliciting groups involved in the expression of silent BGCs is REEs, which have recently been implicated for the production of diverse SMs, including antibiotics, pigments, mycotoxins and phytotoxins [64]. Since REEs are distributed far and wide through the world, microorganisms may have assimilated the ability to respond to minute levels of these elements as an “abiotic” stress over their long evolutionary history, probably as a means of adapting to predominant conditions [99]. Consequently, ACT, dactinomycin (**83**), actinomycin, streptomycin and bacilysin (**84**) production by *S. coelicolor*, *S. antibioticus*, *S. parvulus*, *S. griseus* and *B. subtilis* 168, respectively, has been found to be enhanced, upon the addition of scandium (Sc^3+^) [15]. Scandium exerted its eliciting action at the level of transcription of positive pathway-specific regulatory genes, as clarified by the apparent up-regulation of actII-ORF4 applied in *S. coelicolor* cells, with the critical importance of the bacterial alarmone, guanosine-5′-diphosphate 3′-diphosphate, for ACT overproduction [100]. Additionally, it is thought to act via enhancing enzyme production (α-amylase and bacilysin at the transcriptional level) and secondary metabolism, which was also observed upon adding Sc to *B. subtilis* [101]. Recently, a relevant study showed that cerium (Ce), europium (Eu) and yttrium (Y) upregulated the *actII*-*ORF* transcripts, resulting in the alteration of the transcription of 17 genes fitting to the same number of SMs biosynthetic gene clusters in *S. coelicolor* [15]. Recently, lanthanum chloride (LaCl_3_) was tested in 50 *Actinobacterial* strains for their anti-microbial activities against a variety of pathogens, including *C. albicans*, *Clostridium difficile*, *S. aureus*, MRSA and *Pseudomonas aeruginosa*. Fifteen strains attained enhanced antimicrobial properties upon adding LaCl_3_, with a remarkable enhancement of antimycin-type compounds in strain R818, especially the antifungal compound, urauchimycin D (**85**) (Figure 8). In the same context, lanthanum chloride (50 μM) can provoke antibacterial biosynthetic pathways, upon being added to *Promicromonospora kermanensis* DSM 45485, resulting in the production of the antimycin-type compounds [64]. The main benefit of using REEs in culture media for augmenting antibiotic production, is the absence of need for preceding knowledge of genetic engineering on the strains examined. Since REEs are distributed ubiquitously throughout the world, it is possible that microorganisms have acquired the ability to respond to minute levels of such elements over the course of their evolutionary history, probably as a way of adapting their physiology to the prevailing conditions [102].

### 2.12. Heavy Metals

Elicitation with heavy metals is one of the recent innovative techniques used for drug discoveries. The novel angucycline-type antibiotic, (1S,6S)-3-((3S,4R,5E,7E)-8-cyclopropyl-3-hydroxy-6-methyl-2-oxoocta-5,7-dien-4-yl)-7-oxa-3-aza-bicyclo-[4.1.0]heptan-2-one (**86**), was bioformed in response to nickel supplementation by a marine *Streptomyces* [15], and revealed a strong potency against *Bacillus subtilis* [103]. Similarly, the effect of adding cobalt ions (2mM), in optimized Gause’s medium, to the marine *Actinobacteria streptomyces* sp. H-1003, resulted in enterocin enhancement (**87**), the effect that was completely absent at the normal culture conditions [104]. Variant metals with redox functions, such as Fe, Cu, Mn, Co, Ni, Zn, Mo and Mg, affected, directly or indirectly, the key factors for several microbial enzymes involved in the biosynthesis of SMs, with special regulatory effects on the production of ACT and other related antibiotics in many *Actinomycetes* species [105].

### 2.13. Organic Solvents

Ethanol and DMSO have also been used to elicit the biosynthesis of microbial compounds, possibly by the mistranslation or induction of the stress response [106]. Upon addition of DMSO, a three-fold increase was obtained in chloramphenicol and tetracenomycin C (**88**) production by both *S. venezuelae* ATCC 10712 and *S. glaucescens*, as well as a remarkable expression of the antibiotic coding BGCs in *Promicromonospora kermanensis* [107]. Likewise, a two-fold enhancement of thiostrepton (**89**) and a three-fold increase in both chloramphenicol and tetracenomycin C, respectively, were also observed in *S. azureus* ATCC 14921 upon supplementation with 3% (*v*/*v*) DMSO [107]. Moreover, DMSO has been shown to trigger prodigiosin (**90**) production by *S. lividans* and alter the antibiotic profiles of *Bacillus circulans* and *B. polymyxa*, which was detected by LC-MS analysis [107]. Furthermore, *Promicromonospora kermanensis* DSM 45485 showed noteworthy anti-MRSA activity, after inoculation in an ISP2 broth medium containing both DMSO and VPA, which triggered a modification of the chromatin structure and an activation of ACT BGCs [64]. Adding ethanol to *S. venezuelae* ISP5230 in a d-galactose-l-isoleucine production medium, elicited the production of JdB via the induction of a heat shock response in *S. venezuelae* ISP5230, which served as a metabolite precursor, or alteration of membrane permeability [108] (Figure 8).

## 3. Recent Approaches in Activating Silent BGCs

### 3.1. HITES Approach

**Figure 9:**
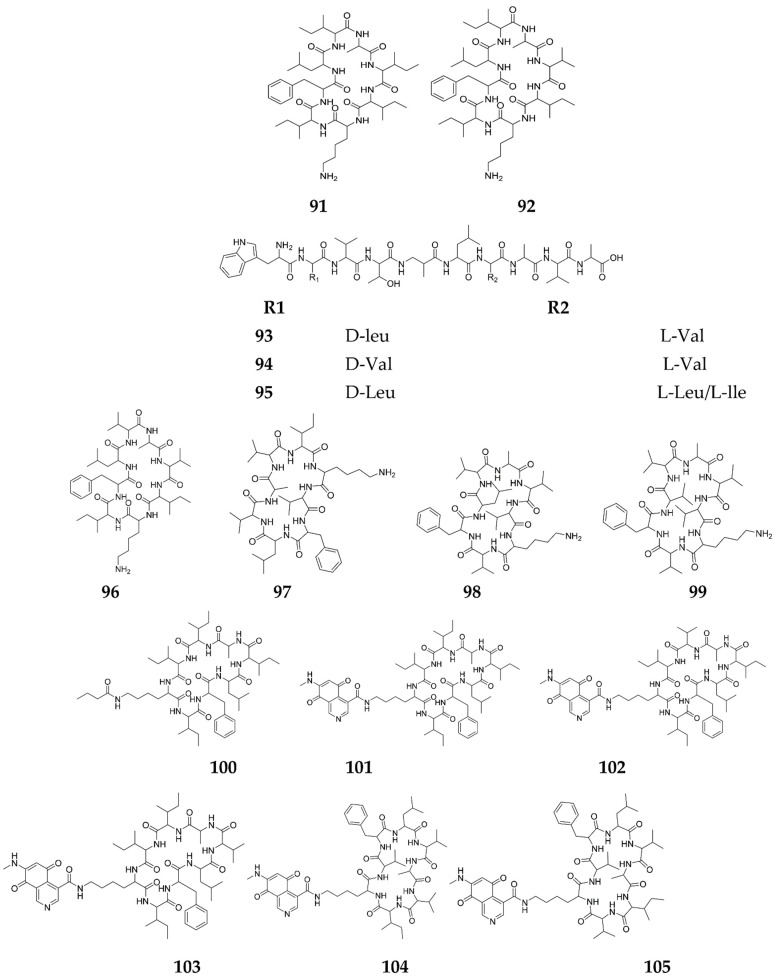

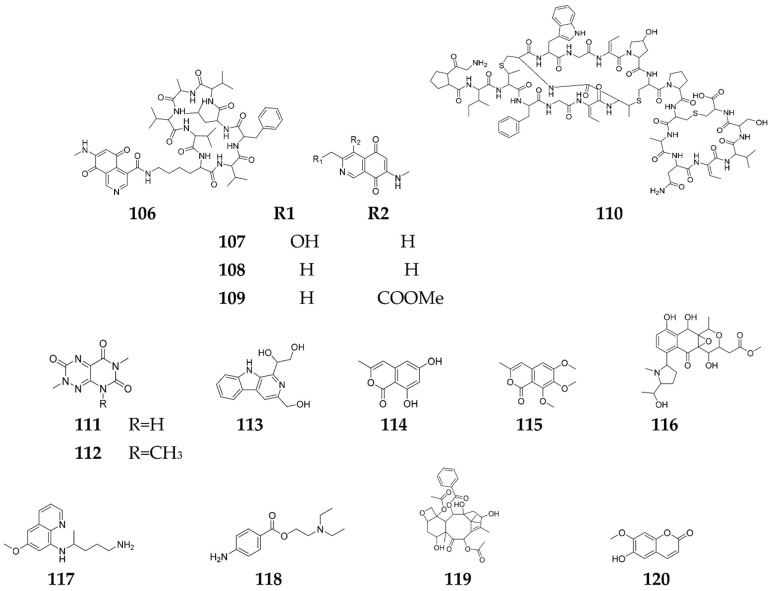
Secondary metabolites elicited by high-throughput screening HiTES.

A recent approach has been designated for the unearthing of small molecule elicitors of silent BGCs, named the chemogenetic high-throughput screening approach, “HiTES”, which has been formerly and successfully applied to the Gram-negative bacterium, *Burkholderia thailandensis*. In HiTES, a reporter gene is presented into a silent BGC of interest to deliver a rapid read-out for expression which utilizes a library of diverse SMs, and the effect of each compound from that library is assayed on the expression of the reporter gene. This is followed by inspecting the detected elicitors from the library, to identify both the production and the induction mechanism of the silent BGC [52,109]. Engaging this approach, activating BGCs can be applicated in a targeted fashion within genetically tractable strains, by changing the elicitors or their concentrations or both. Thus, the level of activation can be tuned and elevated up to ~150-fold, resulting in a significant enhancement of SMs biosynthesis from a given silent BGC. [109]. In a relevant study, six potential antibiotic elicitors were identified through the screening of a commercially-available natural products library (ca. 500 compounds) by HiTES. The most remarkable ones included pleiotropic elicitors, such as ivermectin b1a and etoposide, which attained the maximum surugamide BGC elicitation and changed the SMs profile of *S. albus* J1074—14 [110]. Accordingly, a number of novel SMs were elicited, isolated and characterized, involving surugamides A (**91**), D (**92**), F (**93**), F2 (**94**), F3 (**95**), G–J (**96**–**99**), acyl-surugamide A (**100**) [111], albucyclones A–F (**101**–**106**), albuquinone A (**107**), mansouramycin analog (**108**) and mansouramycin C (**109**) [112], all of which exerted significant cytotoxic activities [113,114]. Acyl-surugamide A exhibited a significant antifungal activity against *Saccharomyces cerevisiae* with an IC_50_ of 3.5 μM, while the mansouramycin analog had a broad anticancer activity, with IC values ranging from 0.250 μM against 36 diverse cancer cell lines [112]. It synergized with sorafenib, a strong inducer of the mitochondrial permeability transition and a talented anticancer drug candidate to induce cell death in A549 cells [115]. These results underline the value of HiTES in the production of bioactive SMs, as was seen with the activation of the silent BGC in *S. albus*, which harbors an excess of silent BGCs, strain J1074 [116], called SurA, that produces surugamides as a result [117]. Investigating the mode of SurA induction led to discovery of a pathway-specific repressor, SurR, which is a pathway-specific transcriptional regulator of the GntR family that silences the *Sur cluster*, all of which explain why little or no products are observed under standard laboratory conditions. Ivermectin b1a and etoposide elicitors induced a 2–2.5-fold downregulation of surR expression, resulting in their stimulatory activities [118]. A study has successfully combined bioactivity assays with (HiTES), to access cryptic, bioactive metabolites in *Saccharopolyspora cebuensis*, with the suppression of *E. coli* growth as a read-out. The results highlighted the identification of a novel lanthipeptide, cebulantin (**110**), elicited with a number of elicitors, including procaine, furosemide and fenofibrate, which showed selectivity against Gram-negative bacterial growth, notably that of diverse *Vibrio* pathogens [119]. HiTES has been fruitfully used to awake BGCs responsible for antibiotic production in *S. hiroshimensis*, via the employment of atenolol, which permits a selective inhibitory activity against *E. coli* and *Acinetobacter baumannii* [120]. Atenolol proved to be a pleiotropic global regulator that affected SM in *S. hiroshimensis*, and enhanced the production of taylorflavins A (**111**) and B (**112**), pyridindolol (**113**) 6,8-dihydroxy-3-methylisocoumarin (**114**), 6,7,8-trimethoxy-3-methylisocoumarin (**115**) and hiroshidine (**116**) [117]. Moreover, *S. lavendulae, S. hiroshimensis* and *Amycolatopsis kerathiniphila*, the producers of streptothricin, keratinimicin, and prodigiosin, respectively, can never produce an antibiotic under normal experimental conditions in an R4 growth medium, but, with the help of an elicitor, they can. In *S. lavendulae* and *S. hiroshimensis*, 7,2′-dimethoxyflavone, isobutylmethylxanthine and methoxyvone induced the synthesis of anti-*B. subtilis* metabolites, including the antimalarial primaquine (**117**) [121] and the local anaesthetic procaine (**118**) [122]. Similarly, aspartame- and tandutinib triggered the bioformation of baccatin III (**119**) and isoscopoletin (**120**) (Figure 9) in *A. keratiniphila*, which resulted in the enhancement of *E. coli* growth. Finally, the metabolomes that most enhanced *E. coli* growth in *S. hiroshimensis*, were elicited by aceclidine, glucosamine and dexchlorpheniramine [117]. Applying the same technique, goadsporin isolated from *Streptomyces* sp. TP-A0584 induced sporulation of many *Streptomyces* species and the production of RED in *S. lividans* [123].

### 3.2. Cumulative Effect of More than One Chemical Elicitor

**Figure 10:**
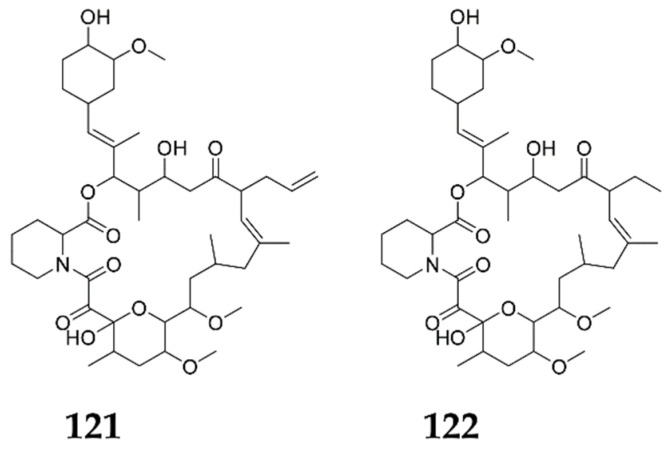
Secondary metabolites elicited by cumulative chemical elicitors and combinatorial engineering.

Nine specific chemical elicitors were assessed for their potential on tacrolimus (**121**) accumulation in *S. tsukubaensis*, the low-yield drug that is clinically approved for prophylaxis against post-transplantation organ rejection (Figure 10). Tacrolimus acts via inhibiting the proliferation of the T cells and the expression of interleukin-2 inside these cells, and it proved to be a potent immunosuppressive alternative to cyclosporine in liver transplantation cases [124]. The investigation results revealed that SB, DMSO and LaCl_3_ can enhance tacrolimus accumulation by more than 30%, and combining DMSO and La increased the yield by 64.7% (303.60 mg/L), compared to the control. A total of 89 SMs, including sugars, amino acids, organic acids, flavonoids and fatty acids, have been detected with a different intracellular metabolism, which is highly correlated with La. Regarding La and SB treatments, diverse groups of resultant amino acids can be created, such as pyruvates (valine and leucine) and aspartates (aspartate, homoserine, lysine and isoleucine) from La treatment, as well as pyruvates (valine and alanine) and aromatic amino acids (tyrosine, phenylalanine and tryptophan) from SB treatment. To obtain an explanation, the impact of amino acids on the tacrolimus production has been studied, following the mechanism of cumulative action of DMSO and La. Different metabolic pathways have been enriched as TCA cycle, amino acid metabolism and benzoate degradation via CoA, compared to the single added chemical elicitor. Although the specific mechanism is still unclear, an obvious response characterization of intracellular metabolism can be detected in the main precursor pathways of tacrolimus synthesis. Additionally, the transition of the methylmalonyl-CoA synthetic pathway in the mutual applications of chemical elicitors has also provided an operative approach to recover tacrolimus, by minimizing the competition for pyruvate and/or acetyl-CoA in tacrolimus biosynthesis. It was clearly concluded that SB, DMSO and REEs (La, Sc, Ho) generated better stimulating effects on tacrolimus accumulation than other solitary chemical elicitors. In a brief conclusion, the augmented metabolic pathways (pentose phosphate and glycolysis pathways) can be greatly enhanced with DMSO treatment, such as methoxymalonyl-CoA, DHCHC, methylmalonyl-CoA, malonyl-CoA and allylmalonyl-CoA. On the other hand, SB treatment triggered PPP and the biosynthesis of unsaturated fatty acids, while the La treatment only enhanced the amino acid metabolism [125].

### 3.3. Combinatorial Engineering Approach

A novel combinatorial engineering approach, depending on the identification of targets that are involved in both the metabolic and transcriptional regulation of many antibiotics, has been totally constructed. Ascomycin (FK520) (**122**) (Figure 10) is a macrocyclic antibiotic that attains antifungal and immunosuppressive potencies, but, unfortunately, it attains relatively low titer and yield, which hampered its pharmaceutical application. Therefore, a rich ascomycin-producing strain, such as *S. hygroscopicus*, was employed and screened employing DMSO, which apparently resulted in good production enhancement. The intracellular metabolic and transcriptional profiles were compared after the introduction of both DMSO and control, where the potential target genes involved in metabolic precursor pathways (zwf and aroA) and other pathways included in the transcriptional regulation (luxR, iclR, fadR and fkbN) have been detected. The peak yield of ascomycin (1258.30 ± 33.49 mg/L), 4.12-fold higher than the control yield (305.60 ± 16.90 mg/L), has resulted from the deviation of carbon flux towards ascomycin accumulation, leading to both metabolic and transcriptional regulation. Combinatory analysis revealed that SB, LaCl_3_ and H_2_O_2_, at their optimum concentrations, improved the ascomycin yield by 33%, 30% and 26%, respectively, while GlcNAc and *γ*-butyrolactones, achieved only minor improvements at the same concentrations. [23].

## 4. Conclusions

**Figure 11:**
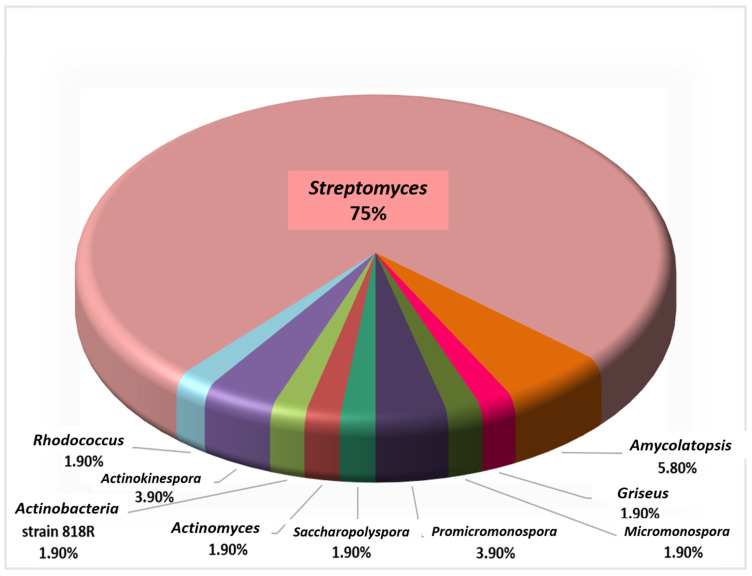
Diversity of actinobacterial genera detected in elicitation studies.

**Figure 12:**
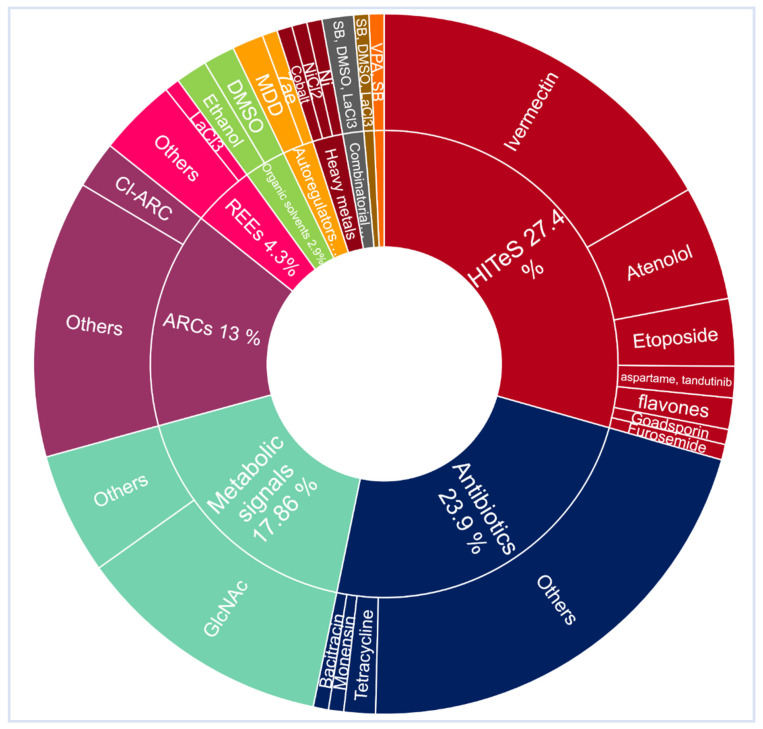
Chemical elicitors used to enhance the production of secondary metabolites.

*Actinomycetes* are considered as rich resources for natural products, with wide range bio-pharmaceutical applications and a rapidly rising number of genome sequencing information, which reveals their potential for SMs biosynthesis. Today, in the light of data obtained from the recently sequenced microbes as well as metagenomic libraries, scientists are aware of the value of untapped existing biosynthetic potential of SMs, yet only the upper tip of the iceberg has been scraped and many valuable natural products are still underexplored. In the same context, the discovery, as well as expansion, of antibiotics is considered as one of the top achievements in the field of curing and prevention of bacterial infections. Regrettably, new infectious diseases and resistant pathogens have been raised at an exceedingly high rate, with the absence of new discoveries of antibiotics that can keep up with this. It is speculated that the existing microbial reservoir needed for these discoveries is exhausted, especially with the increasing rates of antimicrobial resistance that immediately necessitate new approaches to stock-up such antimicrobial drug lines. Thus, it is highly significant to explore new biosynthetic pathways to drive the expression, progress and synthesis of new chemical scaffolds, especially since many of these pathways are observed to be unawake or rarely expressed. Awakening silent BGCs to produce plenty of SMs is considered difficult, but the careful organizing of the strategies described in our mini-review can now be much easier. The mentioned strategies in our mini-review have remarkably increased, especially over the past 5 years, due to genome sequencing and advances in elicitation tools that can make a conspicuous re-invigoration of natural product research. Apart from being potent antimicrobials, many of the chemically elicited SMs mentioned in this review exhibited significant potencies against diverse diseases, with the additional advantage of being pleiotropic regulators. Potent antitumor activities were observed with kigamicin D, mansouramycin, desferrioxamine B, desferrioxamine E, doxorubicin, baumycin, lankacidin, geldanamycin, surfactin lipopeptide 17 and actinomycin D. Actinomycin D, as a potent and clinically approved antitumor drug, also exhibited significant anti-HIV and anti-AIDs activities, similar to geldanamycin and the wide range of antibiotic primaricin, which followed the same behavior. On the other hand, pimaricin, candicidin, urauchimycin D and Acyl-surugamide A showed a remarkable antifungal efficacy, especially Acyl-surugamide A. Interestingly, dynactin, revealed a strong antiparasitic potential against *peronosporomyctes*, which sheds light on its effectiveness to be used as a potential and economic crop protector. Investigating the studied genera, most of the elicited SMs were detected from species belonging to *Streptomyces*, which represent about 75% of the studied genera, in addition to other less studied ones, all of which are illustrated in (Figure 11). Thus, the upcoming efforts for elicitation avenues should be directed towards the rarely studied, yet metabolites-rich, genera as *Actinomyces*, as well as other overlooked genera, as *Nocardia*, *Pseudonocardia*, *Nocardiopsis*, *Salinispora* and *Rhodococcus*. Among the studied techniques (Figure 12), the HiTES technique prevailed where the tested elicitors could enhance the largest portion of the reported SMs with 27.4 %, followed by antibiotics with 23.9 %, while the other groups of chemical elicitors apparently varied. The unique feature of bioactivity HiTES is the high throughput among hundreds of conditions that can be rapidly inspected for cryptic SMs induction. This can be greatly advantageous in directly linking the elicitor to the biosynthesis of the bioactive SM, and also permitting downstream mechanistic investigations that can address the base of the stimulatory activity. In addition, HiTES have recurrently highlighted antibiotics as inducers of silent BGCs, with stimulatory effects at SICs in contrast to inhibitory ones at higher doses. In the same context, atenolol (the antihypertension medication) had a completely different situation as it had no growth-inhibitory effect against many *Streptomyces* species, such as *S. hiroshimensis*. On the other hand, it not only enhanced the production of taylorflavins, but also elicited the production of a variety of SMs, such as pyridindolol, hiroshidine and some other isocoumarins.

In general, the deficient information about the precise physio-ecological signals makes the recent research on elicitation a really challenging mission. This requires interdisciplinary strategies that gather all the data collected from molecular biologists, microbiologists and natural product chemists. Efficient dereplication tools, such as metabolomics and molecular networking, are urgently needed to avoid isolation of previously isolated compounds, and to also appraise the possible novelty of the induced SMs. Furthermore, the structural elucidation of the new elicited SMs via different techniques, such as LC-MS, MS/MS and NMR, will directly lead to discovery of novel chemical scaffolds. Accordingly, future plans addressing the mechanism of induction will deliver additional insights into the regulatory elements that control the secondary metabolism. Apart from the technicalities of using different strategies for decryptification, it is also significant to understand why biosynthetic genes remain cryptic under normal laboratory conditions. Realizing the exact regulation of these cryptic systems would afford the possibility to explore the full potential of BGCs for the new SMs screening. It should be put into consideration that nature is not the only provider of novel natural products; chemical synthesis with modifications of existing moieties also offer potential alternatives. It is worth noting that more efforts should be directed towards exploring the marine environment that suggests novel and chemically rich species, but is underexplored in terms of elicitation.

We could overview the main chemical approaches available for unlocking the biosynthetic potential of *Streptomycetes* with a wide range, easily accessible, low-tech and low-cost methods from HiTES, to the more focused and complex strategies. It is clear that there is no single approach that can be dependably used to unlock every cryptic pathway. We have summarized the techniques that have been developed, to induce secondary metabolism by means of chemical approaches as well as recent advanced ones. We have highlighted current efforts to explore how secondary metabolism is controlled and, therefore, how the silent BGCs can be de-silenced for the aim of next-generation sequencing and advancement in the field of bioinformatics.

In conclusion, the exploration of under-discovered SMs from the veiled sources will ultimately announce solutions for the current crises of “slow moving drug development” and multi-drug-resistant pathogens. In reality, the consortia of such state-of-the-art strategies have reinforced the path for the discovery and development of novel chemical scaffolds to effectively fight multiple drug-resistant pathogens. A great burden faces the scientists now to conduct researches on the hidden features of the microbial treasure of SMs, for the exploration of yet-to be-identified lead bioactive compounds.

## Data Availability

The data presented in this study are available in Supplementary Materials.

## Supplementary Materials

The following supporting information can be found online: Table S1: secondary metabolites detected from different classes of elicitors.
