# Supplementary material for: A Glossary for Chemical Approaches towards Unlocking the Trove of Metabolic Treasures in Actinomycetes
Source: Molecules. 2021 Dec 27;27(1):142. doi: 10.3390/molecules27010142 (PMC8746466; doi:10.3390/molecules27010142)
Supplement: Supplementary file 1 [file molecules-27-00142-s001.zip › molecules-1464484-supplementary.pdf]

(Molecules) Featured Reviews in Natural Products Chemistry

A glossary for chemical approaches towards unlocking the trove of metabolic treasures in *Actinomycetes*

Jianye Zhang [1], Heba Ali Hassan [2,*], Usama Ramadan Abdelmohsen [3,4,*] and Eman Maher Zahran [4,*]

Key Laboratory of Molecular Target & Clinical Pharmacology and the State & NMPA Key Laboratory of Respiratory Disease, School of Pharmaceutical Sciences & the Fifth Affiliated Hospital, Guangzhou Medical University, Guangzhou 511436, China; jianyez@163.com

[2] Department of Pharmacognosy, Faculty of Pharmacy, Sohag University, Sohag 82524, Egypt; heba.ali@pharm.sohag.edu.eg

[3] Department of Pharmacognosy, Faculty of Pharmacy, Minia University, Minia 61519, Egypt

[4] Department of Pharmacognosy, Faculty of Pharmacy, Deraya University, Universities Zone, New Minia City 61111, Minia, Egypt

***** Correspondence: usama.ramadan@mu.edu.eg (U.R.A.); emanzahran84@yahoo.com (E.M.Z.)

***Corresponding authors: emanzahran84@yahoo.com**, **Usama.ramadan@mu.edu.eg, heba.ali@pharm.sohag.edu.eg**

Table S1. Secondary metabolites detected from different classes of elicitors

Studied genera	Chemical elicitor	Produced SMs	Classification	Reference
**Antibiotics**				
Marine *Streptomyces* spp	Bacitracin, tetracycline	Streptophenazines E-H	Elicited	[1]
Marine *Streptomyces* spp	Tetracycline	Streptophenazines A-D	Enhanced	[1]
S. griseorubiginosus strain 574	Monensin 1	Isonitrile antibiotic SF2768	Elicited	[2]
S. griseus	Lincomycin	streptomycin	Enhanced	[2]
S. lividans	Lincomycin	ACT	Enhanced	[3]
Streptomyces Spp	Chloramphenicol	ACT, CDA and piperidamycin	Enhanced	[4]
S. coelicolor M145	Thiosteptone, spectinomycin	RED	Enhanced	[5]
S. venezuelae	Jadomycin B	Jadomycin B	Enhanced	[6]
S. venezuelae	RED	RED	Enhanced	[7]
S. venezuelae ISP5230	DHU and DHR	Jadomycin B	Enhanced	[7]
S. antibioticus	Chlorothricin, and 2 intermediates	Chlorothricin	Enhanced	[8]
S. chartreusis	Calcimycin	Calcimycin	Enhanced	[8]
S. actuosus	Nosiheptide	Nosiheptide	Enhanced	[9]
Amycolatopsis mediterranei,	Rifamycin B	Rifamycin B	Enhanced	[9]
S. venezuelae	JDB	JdB	Enhanced	[10]
S. avermitilis	Avenolide	Avermectin	Enhanced	[10]
S. avermitilis	JdB apramycin, hygromycin B and kanamycin	avermectin	Enhanced	[10]

S. coelicolor	RED	RED, ACT, CDA	Enhanced	[10]
S. albidoflavus S4	Candicidin	Candicidin, antimycin	Enhanced	[11]
S. venezuelae	JadomyciJ	Jadomycin, chloramphenicol	Enhanced	[7]
S. autolyticus CGMCC0516	Geldanamycin	Geldanamycin Elaiophylin	Enhanced	[12]
Streptomyces Spp	ARC2	Erythromycin oligomycin, monensin B, and ACT	Enhanced	[13]
S. coelicolor	ARC2	Germicidins A-C	Enhanced	[13]
S. pristinaespiralis,	ARC2	Desferrioxamine B/E in	Enhanced	[14]
S. peucetius	ARC2	Doxorubicin, baumycin	Enhanced	[14]
S. coelicolor	ARC6	ACT	Enhanced	[14]
S. ghanaensis ATCC 14672,	Cl-ARC 1	Oxohygrolidin	Elicited	[15]
S. hygroscopicus ATCC 53653	Cl-ARC 1	9-methylstreptimidone	Elicited	[16]
WAC0256	Cl-ARC 1	Dynactin,	Elicited	[17]
WAC0256	Cl-ARC 1	Nonactin, monactin, trinactin	Enhanced	[17]
**Histone deacetylase inhibitors**				
S. coelicolor A3 strain M145	Sodium-butyrate, VPA	ACT	Enhanced	[18]
**Hormone-like signalling molecules (autoregulators)**				
S. coelicolor	A-factor	Streptomycin,virginamycin, Valinomycin and methylenomycin	Enhanced	[19]
S. coelicolor	1,4-butyrolactone	Bitespiramycin and validamycin A	Enhanced	[20]
S. virginiae	Butanolide-C	Virginiamycin	Enhanced	[21]
Streptomyces FRI-5	IM-2	Showdomycin, Minimycin	Enhanced	[22]
S. coelicolor	MMF	Methylenomycin A	Enhanced	[21]

S. viridochromogenes	factor 1	Anthracycline	Enhanced	[21]
S. bikiniensis, *S. cyaneofuscatus*	Grafe's	Anthracycline	Enhanced	[21]
S. coelicolor	7ae 1	ACT	Elicited	[19]
S. coelicolor	Hydroxymethylfuran	Methylenomycin	Enhanced	[19]
S. avermitilis	Avenolide	Avermectin	Enhanced	[23]
S. avermitilis	SCB2	Avenolide	Elicited	[23]
S. coelicolor	SCB1	ACT	Enhanced	[24]
S. coelicolor M1152	SCB1–3, SCB4–8	Coelimycin	Enhanced	[25]
S. natalensis	PI factor	Pimaricin	Enhanced	[26]
S. globisporus 1912-B2 and *S. griseus* 1439	MDD 2	Landomycin E and streptomycin	Elicited	[26]
**Chemical signals**				
Micromonospora sp. RV43	GlcNAc	3-formylindole, guaymasol	Enhanced	[27]
Rhodococcus sp. RV157		Bacillibactin, surfactin Lipopeptide 17	Enhanced	[27]
Actinokineospora sp. EG49	GlcNAc	Actinosporins E-H	Elicited	[28]
Actinokineospora spheciospongiae	GlcNAc	Actinosporins C D G, fridamycins H and I	Elicited	[29]
Amycolatopsis alba DSM 44262Δ*abm9*	GlcNAc	Carbamothioic S-acid derivative, kigamicin derivatives	Elicited	[30]
S. coelicolor	Desferrioxamine E	Streptomycin, neomycin, kanamicin	Enhanced	[31]
**Rare earth elements (REEs)**				

S. coelicolor, *S. antibioticus* and *S. parvulus*, *S. griseus* and *B. subtilis* 168	Sc^{+3}	ACT, dactinomycin, actinomycin, streptomycin and bacilysin	Enhanced	[32]
S. coelicolor	Sc^{+3}	ACT	Enhanced	[32]
Actinobacteria strain 818	$LaCl_3$	Urauchimycin D	Elicited	[33]
Promicromonospora kermanensis DSM 45485	La	ACT	Enhanced	[33]
**Heavy metals**				
Marine *streptomycetes*	Ni	Angucycline-type antibiotic	Elicited	[32]
Marine *Streptomyces* sp.	Co	Enterocin	Elicited	[34]
Actinomyces species	$NiCl_2$	ACT	Elicited	[35]
**Organic solvents**				
S. venezuelae ATCC 10712 and *S. glaucescens,*	Ethanol and DMSO	Chloramphenicol and tetracenomycin C	Enhanced	[36]
S. azureus ATCC 14921	DMSO	Thiostrepton, chloramphenicol and tetracenomycin C	Enhanced	[36]
S. lividans	DMSO	Prodigiosin	Elicited	[36]
Promicromonospora kermanensis DSM 45485	DMSO	ACT	Elicited	[18]
S. venezuelae ISP5230	Ethanol	Jadomycin B	Elicited	[37]
S. glaucescens	Ethanol	Tetracenomycin C	Elicited	[18]
**HITES approach**				
S. albus J1074—14	Etoposide	Surugamides (A, D, F, F2, F3 and G-J)	Enhanced	[38]

S. albus J1074	Ivermectin b1a, etoposide	Acyl surugamide A, albucyclones A-F, Albuquinone A	Elicited	[38]
S. albus J1074	Etoposide	Mansouramycin C	Elicited	[39]
S. albus J1074	Etoposide	Mansouramycin analog	Elicited	[39]
Saccharopolyspora cebuensis	Procaine, furosemide, fenofibrate	Cebulantin	Elicited	[40]
S. hiroshimensis		Taylorflavins A and B, pyridindolol, ,6,7,8-trimethoxy-3-methyliso-coumarin and 6,8-dihydroxy-3-methylisocoumarin and hiroshidine	Elicited	[41]
S. lavendulae and S. hiroshimensis	7,2′-dimethoxyflavone, isobutylmethylxanthine, and methoxyvone 2	Primaquine and procaine	Elicited	[42] [43]
S. lividans	Goadsporin	RED	Elicited	[44]
Amycolatopsis. keratiniphila	Aspartame, tandutinib	Baccatin III and isoscopoletin	Elicited	[41]
**Cumulative effect**				
S. tsukubaensis	SB, DMSO and $LaCl_3$	Tacrolimus	Enhanced	[45]
**Combinatorial engineering approach**				
S. hygroscopicus	DMSO	Ascomycin	Enhanced	[46]
S. hygroscopicus	SB, $LaCl_3$, and H_2O_2	Ascomycin	Enhanced	[46]
S. hygroscopicus	GlcNAc and *γ*-butyrolactones	Ascomycin	Enhanced	[46]

References

1. Mitova, M. I.; Lang, G.; Wiese, J.; Imhoff, J. F., Subinhibitory Concentrations of Antibiotics Induce Phenazine Production in a Marine Streptomyces sp. *Journal of natural products* **2008,** 71, (5), 824-827.
2. Amano, S.-i.; Sakurai, T.; Endo, K.; Takano, H.; Beppu, T.; Furihata, K.; Sakuda, S.; Ueda, K., A cryptic antibiotic triggered by monensin. *The Journal of antibiotics* **2011,** 64, (10), 703-703.
3. Zarins-Tutt, J. S.; Barberi, T. T.; Gao, H.; Mearns-Spragg, A.; Zhang, L.; Newman, D. J.; Goss, R. J. M., Prospecting for new bacterial metabolites: a glossary of approaches for inducing, activating and upregulating the biosynthesis of bacterial cryptic or silent natural products. *Natural product reports* **2016,** 33, (1), 54-72.
4. Tanaka, Y.; Izawa, M.; Hiraga, Y.; Misaki, Y.; Watanabe, T.; Ochi, K., Metabolic perturbation to enhance polyketide and nonribosomal peptide antibiotic production using triclosan and ribosome-targeting drugs. *Applied Microbiology and Biotechnology* **2017,** 101, (11), 4417-4431.
5. Wang, H.; Zhao, G.; Ding, X., Morphology engineering of Streptomyces coelicolor M145 by sub-inhibitory concentrations of antibiotics. *Scientific Reports* **2017,** 7, (1), 1-11.
6. Zhang, Y.; Pan, G.; Zou, Z.; Fan, K.; Yang, K.; Tan, H., JadR*-mediated feed-forward regulation of cofactor supply in jadomycin biosynthesis. *Molecular Microbiology* **2013,** 90, (4), 884-897.
7. Wang, L.; Tian, X.; Wang, J.; Yang, H.; Fan, K.; Xu, G.; Yang, K.; Tan, H., Autoregulation of antibiotic biosynthesis by binding of the end product to an atypical response regulator. *Proceedings of the National Academy of Sciences* **2009,** 106, (21), 8617-8622.
8. Li, Y.; Li, J.; Tian, Z.; Xu, Y.; Zhang, J.; Liu, W.; Tan, H., Coordinative modulation of chlorothricin biosynthesis by binding of the glycosylated intermediates and end product to a responsive regulator ChlF1. *Journal of Biological Chemistry* **2016,** 291, (10), 5406-5417.
9. Li, J.; Li, Y.; Niu, G.; Guo, H.; Qiu, Y.; Lin, Z.; Liu, W.; Tan, H., NosP-regulated nosiheptide production responds to both peptidyl and small-molecule ligands derived from the precursor peptide. *Cell chemical biology* **2018,** 25, (2), 143-153. e4.
10. Kong, D.; Wang, X.; Nie, J.; Niu, G., Regulation of Antibiotic Production by Signaling Molecules in Streptomyces. *Frontiers in Microbiology* **2019,** 10, (2927).
11. Xiang, J.; Zhang, J.; Cai, X.; Yang, F.; Zhu, W.; Zhang, W.; Cai, M.; Yu, Z.; Li, X.; Wu, T., Bilobalide protects astrocytes from oxygen and glucose deprivation-induced oxidative injury by upregulating manganese superoxide dismutase. *Phytotherapy Research* **2019,** 33, (9), 2329-2336.
12. Jiang, M.; Yin, M.; Wu, S.; Han, X.; Ji, K.; Wen, M.; Lu, T., GdmRIII, a TetR family transcriptional regulator, controls geldanamycin and elaiophylin biosynthesis in Streptomyces autolyticus CGMCC0516. *Scientific reports* **2017,** 7, (1), 1-11.
13. Olano, C.; Lombo, F.; Mendez, C.; Salas, J. A., Improving production of bioactive secondary metabolites in actinomycetes by metabolic engineering. *Metabolic engineering* **2008,** 10, (5), 281-292.
14. Craney, A.; Ozimok, C.; Pimentel-Elardo, S. M.; Capretta, A.; Nodwell, J. R., Chemical perturbation of secondary metabolism demonstrates important links to primary metabolism. *Chemistry & biology* **2012,** 19, (8), 1020-1027.

15. Kretschmer, A.; Dorgerloh, M.; Deeg, M.; Hagenmaier, H., The structures of novel insecticidal macrolides: bafilomycins D and E, and oxohygrolidin. *Agricultural and biological chemistry* **1985,** 49, (8), 2509-2511.
16. Popović-Djordjević, J. B.; Klaus, A. S.; Žižak, Ž. S.; Matić, I. Z.; Drakulić, B. J., Antiproliferative and antibacterial activity of some glutarimide derivatives. *Journal of Enzyme Inhibition and Medicinal Chemistry* **2016,** 31, (6), 915-923.
17. Pimentel-Elardo, S. M.; Sørensen, D.; Ho, L.; Ziko, M.; Bueler, S. A.; Lu, S.; Tao, J.; Moser, A.; Lee, R.; Agard, D., Activity-independent discovery of secondary metabolites using chemical elicitation and cheminformatic inference. *ACS chemical biology* **2015,** 10, (11), 2616-2623.
18. Mohammadipanah, F.; Kermani, F.; Salimi, F., Awakening the Secondary Metabolite Pathways of Promicromonospora kermanensis Using Physicochemical and Biological Elicitors. *Applied Biochemistry and Biotechnology* **2020,** 192, (4), 1224-1237.
19. Corre, C.; Song, L.; O'Rourke, S.; Chater, K. F.; Challis, G. L., 2-Alkyl-4-hydroxymethylfuran-3-carboxylic acids, antibiotic production inducers discovered by Streptomyces coelicolor genome mining. *Proceedings of the National Academy of Sciences* **2008,** 105, (45), 17510-17515.
20. Gao, X.; Wang, Y.; Chu, J., A preliminary study on the impact of exogenous A-Factor analogue 1,4-butyrolactone on stimulating bitespiramycin biosynthesis. *Bioprocess and Biosystems Engineering* **2019,** 42, (12), 1903-1913.
21. Novakova, R.; Rehakova, A.; Kutas, P.; Feckova, L.; Kormanec, J., The role of two SARP family transcriptional regulators in regulation of the auricin gene cluster in Streptomyces aureofaciens CCM 3239. *Microbiology* **2011,** 157, (6), 1629-1639.
22. Hashimoto, K.; Nihira, T.; Sakuda, S.; Yamada, Y., IM-2, a butyrolactone autoregulator, induces production of several nucleoside antibiotics in Streptomyces sp. FRI-5. *Journal of fermentation and bioengineering* **1992,** 73, (6), 449-455.
23. Kitani, S.; Miyamoto, K. T.; Takamatsu, S.; Herawati, E.; Iguchi, H.; Nishitomi, K.; Uchida, M.; Nagamitsu, T.; Omura, S.; Ikeda, H., Avenolide, a Streptomyces hormone controlling antibiotic production in Streptomyces avermitilis. *Proceedings of the National Academy of Sciences* **2011,** 108, (39), 16410-16415.
24. Yang, Y.-H.; Kim, T.-W.; Park, S.-H.; Lee, K.; Park, H.-Y.; Song, E.; Joo, H.-S.; Kim, Y.-G.; Hahn, J.-S.; Kim, B.-G., Cell-free Escherichia coli-based system to screen for quorum-sensing molecules interacting with quorum receptor proteins of Streptomyces coelicolor. *Applied and environmental microbiology* **2009,** 75, (19), 6367-6372.
25. Sidda, J. D.; Poon, V.; Song, L.; Wang, W.; Yang, K.; Corre, C., Overproduction and identification of butyrolactones SCB1–8 in the antibiotic production superhost Streptomyces M1152. *Organic & biomolecular chemistry* **2016,** 14, (27), 6390-6393.
26. Matselyukh, B.; Mohammadipanah, F.; Laatsch, H.; Rohr, J.; Efremenkova, O.; Khilya, V., N-methylphenylalanyl-dehydrobutyrine diketopiperazine, an A-factor mimic that restores antibiotic biosynthesis and morphogenesis in Streptomyces globisporus 1912-B2 and Streptomyces griseus 1439. *The Journal of Antibiotics* **2015,** 68, (1), 9-14.
27. Meena, K. R.; Sharma, A.; Kanwar, S. S., Antitumoral and Antimicrobial Activity of Surfactin Extracted from Bacillus subtilis KLP2015. *International Journal of Peptide Research and Therapeutics* **2020,** 26, (1), 423-433.

28. Dashti, Y.; Grkovic, T.; Abdelmohsen, U. R.; Hentschel, U.; Quinn, R. J., Actinomycete Metabolome Induction/Suppression with N-Acetylglucosamine. *Journal of natural products* **2017,** 80, (4), 828-836.
29. Tawfike, A.; Attia, E. Z.; Desoukey, S. Y.; Hajjar, D.; Makki, A. A.; Schupp, P. J.; Edrada-Ebel, R.; Abdelmohsen, U. R., New bioactive metabolites from the elicited marine sponge-derived bacterium Actinokineospora spheciospongiae sp. nov. *AMB Express* **2019,** 9, (1), 12.
30. Zahran, E. M.; Abdelmohsen, U. R.; Ayoub, A. T.; Salem, M. A.; Khalil, H. E.; Desoukey, S. Y.; Fouad, M. A.; Kamel, M. S., Metabolic profiling, histopathological anti-ulcer study, molecular docking and molecular dynamics of ursolic acid isolated from Ocimum forskolei Benth.(family Lamiaceae). *South African Journal of Botany* **2020,** 131, 311-319.
31. Yamanaka, K.; Oikawa, H.; Ogawa, H.-o.; Hosono, K.; Shinmachi, F.; Takano, H.; Sakuda, S.; Beppu, T.; Ueda, K., Desferrioxamine E produced by Streptomyces griseus stimulates growth and development of Streptomyces tanashiensis. *Microbiology* **2005,** 151, (9), 2899-2905.
32. Abdelmohsen, U. R.; Grkovic, T.; Balasubramanian, S.; Kamel, M. S.; Quinn, R. J.; Hentschel, U., Elicitation of secondary metabolism in actinomycetes. *Biotechnology advances* **2015,** 33, (6), 798-811.
33. Xu, D.; Han, L.; Li, C.; Cao, Q.; Zhu, D.; Barrett, N. H.; Harmody, D.; Chen, J.; Zhu, H.; McCarthy, P. J., Bioprospecting deep-sea actinobacteria for novel anti-infective natural products. *Frontiers in microbiology* **2018,** 9, 787.
34. Hassan, S. S.; Shah, S. A. A.; Pan, C.; Fu, L.; Cao, X.; Shi, Y.; Wu, X.; Wang, K.; Wu, B., Production of an antibiotic enterocin from a marine actinobacteria strain H1003 by metal-stress technique with enhanced enrichment using response surface methodology. *Pak. J. Pharm. Sci* **2017,** 30, (1), 313-324.
35. Haferburg, G.; Groth, I.; Möllmann, U.; Kothe, E.; Sattler, I., Arousing sleeping genes: shifts in secondary metabolism of metal tolerant actinobacteria under conditions of heavy metal stress. *Biometals* **2009,** 22, (2), 225.
36. Chen, G.; LI, X.; Waters, B.; Davies, J., Enhanced production of microbial metabolites in the presence of dimethyl sulfoxide. *The Journal of antibiotics* **2000,** 53, (10), 1145-1153.
37. Pettit, R. K., Small-molecule elicitation of microbial secondary metabolites. *Microbial biotechnology* **2011,** 4, (4), 471-478.
38. Li, X.; Wu, Y.; Zhang, W.; Gong, J.; Cheng, Y., Pre-conditioning with tanshinone IIA attenuates the ischemia/reperfusion injury caused by liver grafts via regulation of HMGB1 in rat Kupffer cells. *Biomedicine & Pharmacotherapy* **2017,** 89, 1392-1400.
39. Seyedsayamdost, M. R., Toward a global picture of bacterial secondary metabolism. *Journal of industrial microbiology & biotechnology* **2019,** 46, (3-4), 301-311.
40. Moon, K.; Xu, F.; Seyedsayamdost, M. R., Cebulantin, a Cryptic Lanthipeptide Antibiotic Uncovered Using Bioactivity-Coupled HiTES. *Angewandte Chemie* **2019,** 131, (18), 6034-6038.
41. Moon, K.; Xu, F.; Zhang, C.; Seyedsayamdost, M. R., Bioactivity-HiTES unveils cryptic antibiotics encoded in actinomycete bacteria. *ACS chemical biology* **2019,** 14, (4), 767-774.

42. Camarda, G.; Jirawatcharadech, P.; Priestley, R. S.; Saif, A.; March, S.; Wong, M. H. L.; Leung, S.; Miller, A. B.; Baker, D. A.; Alano, P.; Paine, M. J. I.; Bhatia, S. N.; O'Neill, P. M.; Ward, S. A.; Biagini, G. A., Antimalarial activity of primaquine operates via a two-step biochemical relay. *Nature Communications* **2019,** 10, (1), 3226.
43. Schmidt, R. M.; Rosenkranz, H. S., Antimicrobial activity of local anesthetics: lidocaine and procaine. *The Journal of infectious diseases* **1970**, 597-607.
44. Onaka, H., Novel antibiotic screening methods to awaken silent or cryptic secondary metabolic pathways in actinomycetes. *The Journal of antibiotics* **2017,** 70, (8), 865-870.
45. Kelly, P. A.; Burckart, G. J.; Venkataramanan, R., Tacrolimus: a new immunosuppressive agent. *American journal of health-system pharmacy : AJHP : official journal of the American Society of Health-System Pharmacists* **1995,** 52, (14), 1521-35.
46. Wang, C.; Wang, J.; Yuan, J.; Jiang, L.; Jiang, X.; Yang, B.; Zhao, G.; Liu, B.; Huang, D., Generation of Streptomyces hygroscopicus cell factories with enhanced ascomycin production by combined elicitation and pathway-engineering strategies. *Biotechnology and bioengineering* **2019,** 116, (12), 3382-3395.
